# A novel method for identifying key genes in macroevolution based on deep learning with attention mechanism

**DOI:** 10.1038/s41598-023-47113-9

**Published:** 2023-11-13

**Authors:** Jiawei Mao, Yong Cao, Yan Zhang, Biaosheng Huang, Youjie Zhao

**Affiliations:** 1 College of Big Data and Intelligent Engineering, Southwest Forestry University, Kunming, 650224 China; 2 College of Mathematics and Physics, Southwest Forestry University, Kunming, 650224 China

**Keywords:** Computational biology and bioinformatics, Evolution

## Abstract

Macroevolution can be regarded as the result of evolutionary changes of synergistically acting genes. Unfortunately, the importance of these genes in macroevolution is difficult to assess and hence the identification of macroevolutionary key genes is a major challenge in evolutionary biology. In this study, we designed various word embedding libraries of natural language processing (NLP) considering the multiple mechanisms of evolutionary genomics. A novel method (IKGM) based on three types of attention mechanisms (domain attention, kmer attention and fused attention) were proposed to calculate the weights of different genes in macroevolution. Taking 34 species of diurnal butterflies and nocturnal moths in Lepidoptera as an example, we identified a few of key genes with high weights, which annotated to the functions of circadian rhythms, sensory organs, as well as behavioral habits etc. This study not only provides a novel method to identify the key genes of macroevolution at the genomic level, but also helps us to understand the microevolution mechanisms of diurnal butterflies and nocturnal moths in Lepidoptera.

## Introduction

Traces of macroevolution are widespread in nature, such as the aquatic to terrestrial evolution of vertebrates^1–3^, the warm-bloodedness of mammals^4^, the origin of bird wings^5^, the origin of animal taste organs^6^, etc. During the evolutionary process that occurs over long time scales, macroevolution was generally the result of synergistic action of complex molecular mechanisms at the genome level in addition to environmental factors^7,8^. However, among these possible molecular mechanisms, it is difficult to identify the key genes that drive the macroevolution of taxa. Therefore, it has become an important but unsolved problem in evolutionary biology to quantify the weight of key functional genes that cause macroevolution of ancestral species.

Previous studies^9–11^ have shown that polyploidization (or whole-genome duplication, WGD) is the major driver for species formation and macroevolution in plants. However, WGD events are relatively rare and have only been found in a few animal taxa, such as euryhaline fishes^12^ and Arachnida^13,14^. The molecular mechanisms involved in animal macroevolution are mainly as follows: (1) Contraction and expansion of gene families; for example, the emergence of epithelial tubular organs in vertebrates was associated with contraction and expansion of the Claudins gene family^15^; the evolution of functional plough nose organs in mammals was associated with contraction and expansion of the OR gene family^16^. (2) Selective evolution of genes in response to environmental stress; for example, the evolutionary rate of genes involved in energy metabolism, low-oxygen adaptation and skeletal development was significantly faster in ground tits that occur at high-altitudes compared to the closely related species^17^; studies of visual proteins in Lepidopteran insects showed that visual genes associated with brighter environments evolved faster and were under positive selection in insects from diurnal taxa^18^. (3) Structure variation in genomes, such as the evolution of butterfly wing mimicry duo to the inversions^19^.

The above-mentioned studies on the molecular mechanisms of macroevolution are mainly based on traditional bioinformatics and statistical methods^20–22^. Considering the complex mechanisms of multiple key genes in the process of macroevolution, it is a challenge to systematically identify the key genes of macroevolution at the genome level. In order to obtain new knowledge from huge genomic data, machine learning (ML) has become a widely used and successful approach^23–25^. The correct performance of traditional ML algorithms relies heavily on data representations called features, and different features often need to be constructed for different task objectives. Moreover, deep learning (DL), a subfield of ML, can automatically learn features and patterns from data without the need for manual feature engineering. DL has been applied to various aspects of biological research and has shown powerful capabilities^26–30^, such as the analysis of gene expression data, and DNA and protein sequence data using natural language processing (NLP) related techniques with recurrent neural networks as the cornerstone. Despite the excellent results achieved by DL in several areas of bioinformatics, the inference process of DL is agnostic. In some bioinformatics scenarios, interpretable inference processes are often as important or even more important than excellent results. The attention mechanism (AM)^31^ can compute different weights for different parts of the training sample during the training process. During the inference step without additional computation, these weights are generated and considered as the importance of that part to the model. The part with high weight was always focused on in the training process of model, and this can explain the inference process of DL. Previous studies showed that AM has been applied in the more and more fields of bioinformatics, such as the prediction of enhancers^32^, and the prediction of protein interactions^33^, etc. However, it is still a challenge to use AM to identify the different weights of key genes in the macroevolution of taxa.

In this paper, we develop IKGM, a method based on deep learning with attention mechanisms for identifying key genes in the macroevolution of biological taxa, which allows attaching different weights to genes to characterize the importance of these genes in macroevolutionary processes. Using 34 species of diurnal butterflies and nocturnal moths as an example, we used IKGM to mine the key genes with high weights and performed KEGG enrichment analysis based on these genes. These results should help us to understand the mechanisms of macroevolution in Lepidoptera.

## Materials and methods

### Data source

All the protein sequences of 34 Lepidoptera species were downloaded from *InsectBase*^34^ and protein-coding genes were used as original samples. These species were labeled into two groups (nocturnal or diurnal) according to the diel behavior information in previous studies^18,35^ (Supplementary-file2: Table S1). The proteins of these species were annotated to obtain the domain information by Pfam database (http://pfam.xfam.org/).

### Pipeline of IKGM

**Figure 1:**
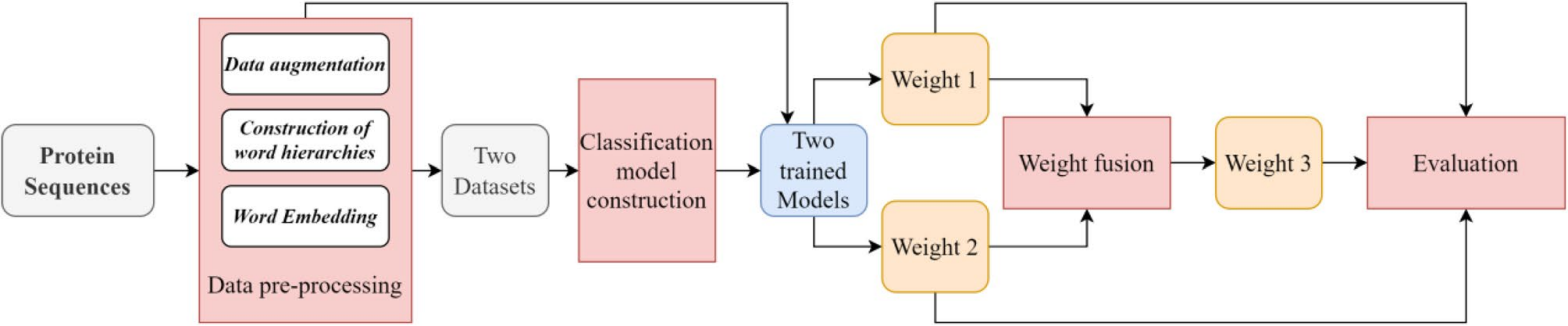
Pipeline of IKGM in this study. The red rectangles (Data pre-processing, Classification model construction, Weights fusion, and Evaluation) indicate the four key components of IKGM. Data pre-processing part consists of three main sub-modules, which use NLP technology to complete the pre-processing of raw protein sequences and feature construction. Classification model construction is the modeling of macroscopic evolutionary processes using deep learning and hierarchical attention mechanisms. Weight fusion is the process of fusing two different sets of gene weights together using a certain computational strategy (AM), where the gene weights represent the importance of the gene in the classification process, also known as the contribution to macroevolution. Evaluation is the process of annotation and KEGG enrichment for these genes with high weights.

In this study, the diel behavior information of 34 Lepidoptera species is used as the classification labels of experimental samples. The macroevolution phenomenon of nocturnal moths and diurnal butterflies is modeled as a classification problem of protein sequences. NLP was used to construct the word embedding libraries based on these sequences, and then AM is added to the classification network to compute the weight of different genes in the classification process. The pipeline of this paper mainly consists of four important parts (Fig. 1): Data pre-processing, Classification Model, Weights calculation, and Evaluation. In this study, three types of attention mechanisms (domain attention, kmer attention and fused attention) were developed to calculate the weights (weight 1, weight 2 and weight 3 in Fig. 1) of different genes. The details of each part are described in the following subsections. In addition, some important symbols in the method are shown in Table 1.

**Table 1:** Description of the meaning of some important symbols.

Symbols	Description
$HAN$	Hierarchical Attention Network for genome classification
${\alpha }_{i,t}$	Attention score of the $t$th word in the $i$th sentence in the hierarchical attention network
${\alpha }_{i}$	Attention score of the $i$th sentence in the hierarchical attention network
$v$	Hierarchy of words
${PAS}_{v,s}$	Protein attention scores for species $s$ when using word hierarchy $v$
${GAS}_{v,T}$	Gene attention scores for taxon $T$ when using word hierarchy $v$
${DAS}_{v}$	Attention scores of genes causing differences between the two extant taxa when using word hierarchy $v$

### Data pre-processing

#### Data augmentation

Considering the prevalence of single nucleotide polymorphisms (SNP), insertion and deletion (InDel) and structural variants (SV) in different populations for each species, the original samples of 34 Lepidoptera species were expanded by simulating sequence variants to meet the sample requirements of the deep learning algorithm. In addition, we refer to the Neutral Theory of Molecular Evolution (Neutral Theory) proposed by Motoo Kimura, and attempt to make random small-scale mutations without selection bias while not changing the overall phenology of the species. We performed the simulation in several ways: (1) gene rearrangement, we divide the entire genome into multiple parts and perform random interchanges between the multiple parts without changing the gene order within each part; and (2) sequence mutations, i.e., random mutations of amino acids in a portion of the protein sequence, with an overall frequency of mutations of less than 1% of the genome. In addition, considering that the number of samples from the two taxa is uneven, the data are augmented separately for the two categories of genomes. In order to ensure that the mutated genomes are as diverse as possible, the number of amplifications is kept consistent for each original genome, the number of genomes before and after augmentation of each taxon and the number of each individual genome augmented by mutation (as shown in Supplementary-file2: Table S2).

#### Construction of word hierarchies

In natural language, multiple words are arranged in a certain word order to form a sentence with semantic meaning, while multiple sentences arranged in a certain order can form a text with rich semantic meaning. By analogy with natural language, the protein sequences of a single species can be considered as a text, while a single protein sequence can be considered as a sentence. However, word hierarchies are not clearly represented in proteins, and using an amino acid character as a word not only does not reflect the molecular mechanisms that may lead to macroevolution, but also leads to excessively long sentences. To address this problem and construct biologically meaningful word hierarchies to characterize the evolutionary mechanisms at different scales, we propose two methods for constructing word hierarchies:

1. Word hierarchy construction based on domain name (*v* = *1*);
  Given that contractions and expansions of gene family often lead to quantitative differences in functional domains, and Pfam annotation of the original samples was performed. The annotated functional domain names are then used as word hierarchies to express contractions and expansions of gene families that may occur, as shown in Fig. 2a.

**Figure 2:**
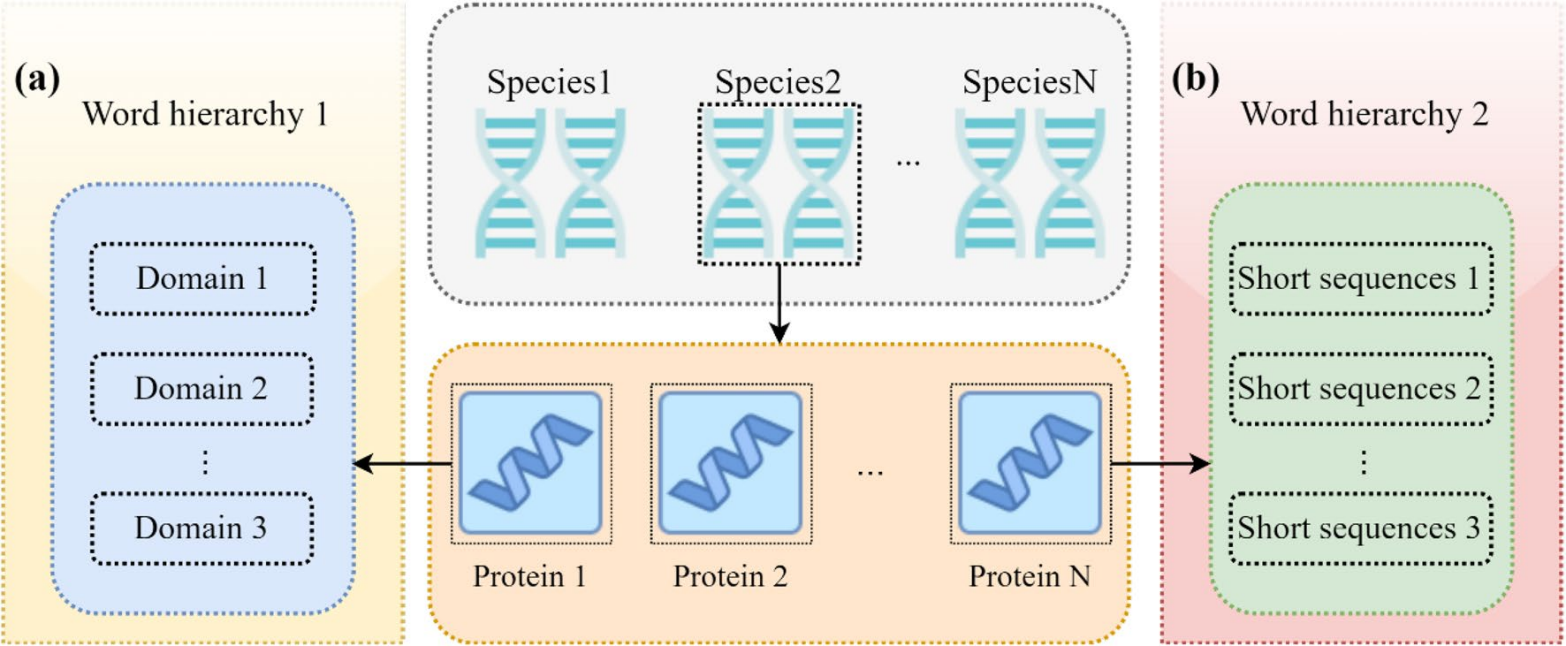
Word hierarchy construction. (**a**) Use the name of the Pfam functional domain contained in a protein as a word hierarchy to characterize the protein. (**b**) Short sequences obtained using the variant-based *kmer* method were used as word hierarchies to characterize the proteins.
2. Word hierarchy construction based on variant kmer (*v* = *2*);
  The selective evolution of genes is often reflected in the sequence differences of amino acids. So, a sliding window is performed on all sequences according to the fixed length* k* and frequency statistics are performed on the obtained *kmer.* Then the high-frequency (greater than the quartile) *kmer* is selected as the segment marker, segment operation is performed on all the protein sequences, and all unequal short sequences obtained were used as word hierarchies to characterize the possible selective evolution of genes (as shown in Supplementary -file 1: Fig. S1). These short sequences can be viewed as unequal short peptides, as shown in Fig. 2b.

#### Word embedding

As mentioned in Sect. “Introduction”, in traditional machine learning methods, it is often necessary to require feature engineering, which is usually based on a statistical approach to the original sequence. Their traditional features require manual construction, such as counting the spectrum of *kmer* in the sequence as a feature of the input sequence^36^. However, this approach does not fully represent all the information contained in the original sequence in particular, nor does it reflect the key contextual relationships. With the rise of deep learning, feature methods now focus more on the original sequence itself by directly encoding the original sequence to vectorize input features, such as one-hot encoding. However, the one-hot encoded vector is too sparse and does not express the correlation between the meanings of words in the original sequence. Unlike one-hot coding, a technique called word embedding captures the semantic association between words and can help obtain a better and more specific representation of sequence features. In this paper, for the data represented by the two types of word hierarchies mentioned above, the word embedding pre-training is performed using the *Skip-gram* algorithm^37^ to obtain a vector representation of sequences with embedding dimension 200. The internal words of each protein sequence are replaced with the word vector representation obtained from the pre-trained model, which is converted into a feature matrix by concatenating all word embedding vectors in that protein sequence. Similarly, the feature matrix corresponding to each protein sequence is concatenated to obtain a complete vector representation of all protein sequences in a species.

### Classification model construction and weight calculation

**Figure 3:**
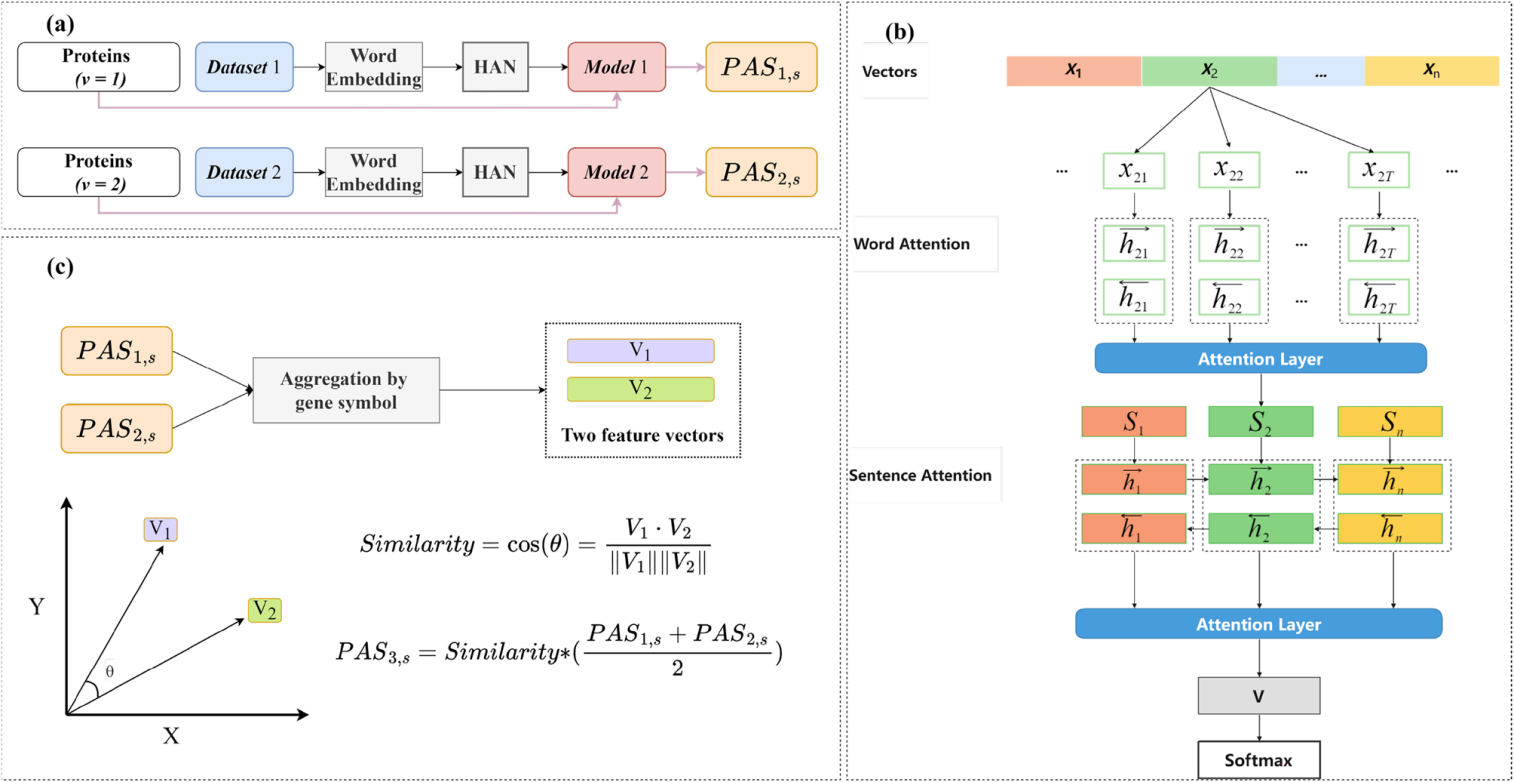
Schematic diagram of classification model and weight calculation. (**a**) The main process of classification, after data pre-processing using two type word hierarchical representations of two data sets were pre-trained with word embedding through a hierarchical attention classification network to obtain two classification models, and the original protein sequences of each specie were input into the trained classification model to obtain the domain attention scores (*PAS*_1*,s*_) and kmer attention scores (*PAS*_2*,s*_). (**b**) The network architecture of the hierarchical attention classification network (HAN) with two attention layers added to the basic classification network. (**c**) Fused attention scores (*PAS*_3*,s*_) based on two sets of weights (*PAS*_1*,s*_ and *PAS*_2*,s*_).

Considering the different protein information using different word hierarchy representations, as shown in Fig. 3a, and the two classification networks were trained. After that, the two representations of the original protein sequences of each species were input to the two trained models to obtain the domain attention scores (*PAS*_1*,s*_) and kmer attention scores (*PAS*_2*,s*_) for each species. Considering the hierarchical structure of the samples and the need for attention scores, this paper uses a hierarchical attention classification network to capture the weights at different hierarchies. The architecture of the hierarchical attention classification network is shown in Fig. 3b. The two sets of *PAS*_*v,s*_(*PAS*_*1,s*_ and *PAS*_*2,s*_) were fused to obtain the fused attention scores (*PAS*_3*,s*_) using the self-attention mechanism to reflect the variation mechanisms captured by different word hierarchies simultaneously, as shown in Fig. 3c. The details of the hierarchical attention classification network and the self-attention fusion module will be described in the next subsections.

#### Hierarchical attention classification network

In the classification process, the abstract hidden layer representation of each data segment is obtained by passing the data through *BiLSTM*. The *x*_*i,t*_ is the *i*th word vector in the sequence at time *t* , and the bidirectional $\overrightarrow{{h}_{i,t}}$ and $\overleftarrow{{h}_{i,t}}$ represent the forward and backward hidden layer states of the $\overrightarrow{{h}_{i,t-1}}$ and $\overleftarrow{{h}_{i,t+1}}$ in the *BiLSTM* (Eqs. (1), (2)). The bidirectional information is integrated to obtain the bidirectional hidden layer state ${h}_{i,t}$(Eq. (3)).

1 $$\begin{array}{c}\overrightarrow{{h}_{i,t}}=\overrightarrow{LSTM}\left({x}_{i,t},\overrightarrow{{h}_{i,t-1}}\right)\end{array}$$

2 $$\begin{array}{c}\overleftarrow{{h}_{i,t}}=\overleftarrow{LSTM}\left({x}_{i,t},\overleftarrow{{h}_{i,t+1}}\right)\end{array}$$

3 $$\begin{array}{c}{h}_{i,t}=\left[\overrightarrow{{h}_{i,t}},\overleftarrow{{h}_{i,t}}\right]\end{array}$$

The bidirectional information of the word vector *x*_*i,t*_ is combined into ${h}_{i,t}$ and it is inputted into an MLP for activation to obtain the nonlinear hidden layer representation ${u}_{i,t}$ (Eq. (4), ${h}_{i,t}^{\intercal }\mathrm{represents\, the\, transpose\, of\,} {h}_{i,t}$) of the word vector *x*_*i,t*_. In general, different words have different emotional color biases for the sentence, that is, they have different importance for a sentence, as shown in Supplementary-file 1: Fig. S2a, in order to make the BiLSTM focus on important words, we design the attention mechanism based on word vectors, as formulated in Eqs. (5) and (6).

4 $$\begin{array}{c}{u}_{i,t}=tanh\left({W}_{w}{h}_{i,t}^{\intercal }+{b}_{w}\right)\end{array}$$

5 $$\begin{array}{c}{\alpha }_{i,t}=\frac{exp\left({w}_{w}{u}_{i,t}\right)}{{\sum }_{t}exp\left({w}_{w}{u}_{i,t}\right)}\end{array}$$

6 $$\begin{array}{c}{s}_{i}={\sum }_{t}{\alpha }_{i,t}{h}_{i,t}\end{array}$$

where ${W}_{w}$ and ${b}_{w}$ are the weight matrix and the bias term in the tanh function (Eqs. (4)), respectively. To better represent the word importance represented by the attention weights, we normalized the attention weights (Eqs. (5)) where ${w}_{w}$ represents the vector of the context of ${u}_{i,t}$, and ${\alpha }_{i,t}$ denotes the attention weight of the word vector *x*_*i,t*_ . The sentence vector ${s}_{i}$ represents the weight sum of the product of the word weights and the hidden layer state information ${h}_{i,t}$. Similar to word-level attention, different sentences have different importance for a text, as shown in Supplementary-file 1: Fig. S2b, sentence-level attention is designed so that the classification network focuses on the important sentences based on the attention weights.

7 $$\begin{array}{c}\overrightarrow{{h}_{i}}=\overrightarrow{LSTM}\left({s}_{i},\overrightarrow{{h}_{i-1}}\right)\end{array}$$

8 $$\begin{array}{c}\overleftarrow{{h}_{i}}=\overleftarrow{LSTM}\left({s}_{i},\overleftarrow{{h}_{i+1}}\right)\end{array}$$

9 $$\begin{array}{c}{h}_{i}=\left[\overrightarrow{{h}_{i}},\overleftarrow{{h}_{i}}\right]\end{array}$$

where $\overrightarrow{{h}_{i}}$ and $\overleftarrow{{h}_{i}}$ are the forward and backward implicit variables of $\overrightarrow{{h}_{i-1}} \mathrm{and }\overleftarrow{{h}_{i+1}}$ for the *i*th sentence in the text, respectively. ${h}_{i}$ is the implicit variable for the $i$ th sentence in both directions. Similar to the attention mechanism at the word level, a sentence attention mechanism is designed for the sentence level, and it is calculated as follows:

10 $$\begin{array}{c}{u}_{i}=tanh\left({W}_{s}{h}_{i}^{\intercal }+{b}_{s}\right)\end{array}$$

11 $$\begin{array}{c}{\alpha }_{i}=\frac{exp\left({w}_{s}{u}_{i}\right)}{{\sum }_{i}exp\left({w}_{s}{u}_{i}\right)}\end{array}$$

12 $$\begin{array}{c}P={\sum }_{t}{\alpha }_{i}{h}_{i}\end{array}$$

where ${W}_{s}$ and ${b}_{s}$ are the weight matrix and bias vector of the tanh function at the sentence level, respectively. ${w}_{s}$ represents the vector of the context of ${u}_{i}$, ${\alpha }_{i}$ is the normalized attention weight of the $i$ th sentence. And $P$ is the weight sum of all sentences in the text(genome) and the attention of the sentence is its weight. Finally, $P$ is fed to a fully connected layer to calculate the output classification probability $\widehat{y}$.

13 $$\begin{array}{c}\widehat{y}=softmax\left({W}_{c}P+{b}_{c}\right)\end{array}$$

#### Weight acquisition and fusion

The two sets of *PAS*_*v,s*_ (*PAS*_*1,s*_ and *PAS*_*2,s*_) from different v were aggregated according to gene symbols and the aggregation results were considered as two sets of feature vectors as shown in Fig. 3c. The similarity between these two sets of feature vectors is calculated using cosine similarity (Eq. (14)) and use the multiplication of this similarity and the mean of the first two sets of *PAS*_*v,s*_ as PAS_3,s_ (Eq. (15)).

14 $$\begin{array}{c}similarity=cos(\theta )=\frac{{V}_{1}\cdot {V}_{2}}{\Vert {V}_{1}\Vert \Vert {V}_{2}\Vert }\end{array}$$

15 $$\begin{array}{c}{PAS}_{3,s}=similarity *\left(\frac{{PAS}_{1,s} +{PAS}_{2,s}}{2}\right)\end{array}$$

### Evaluation and KEGG enrichment

The normalized *PAS*_*v,s*_ corresponding to protein sequences with the same annotation name are summed as the two total contributions of the protein sequences to the taxon *GAS*_*v,T*_. The normalized *GAS*_*v,T*_ of the two taxa are summed as the total contribution to the classification process(Eq. (16)).

16 $$\begin{array}{c}{DAS}_{v}= \left[normalize\left({GAS}_{\left(v,diurnal\right)}\right)+normalize\left({GAS}_{\left(v,nocturnal\right)}\right)\right]\end{array}$$

In this paper, we ranked the genes according to *DAS*_*v*_ (weight of genes) from high to low, and the top 1% of genes were used as the key genes for the macroevolution of Lepidoptera. In order to explore the difference of high-weight genes between butterflies and moths, we analyzed the evolution of ninaB, GNB1l and eys genes obtained by different types of attention mechanisms (domain attention, kmer attention and fused attention). The phylogeny tree of 18 butterflies and 13 moths was obtained from Timetree (http://www.timetree.org/) (three butterfly species are missing in Timetree). NinaB genes were identified based on the annotation of InsectBase (http://v2.insect-genome.com/). Sequence of GNB1l genes was aligned by MegaX^38^. Domains of eys genes were annotated by Pfam (http://pfam.xfam.org/). To verify the accuracy of the key genes leading to macroevolution in Lepidoptera identified in this paper, the enrichment analysis of the KEGG metabolic pathway and the search of the corresponding gene functions were performed. If the enriched metabolic pathways are significantly associated with differences in Diel behavior or if the functions of certain genes are associated with certain macroscopic phenotypes of the two major taxa, then the approach of this paper is proven to be effective.

## Result

### Data pre-processing results

**Figure 4:**
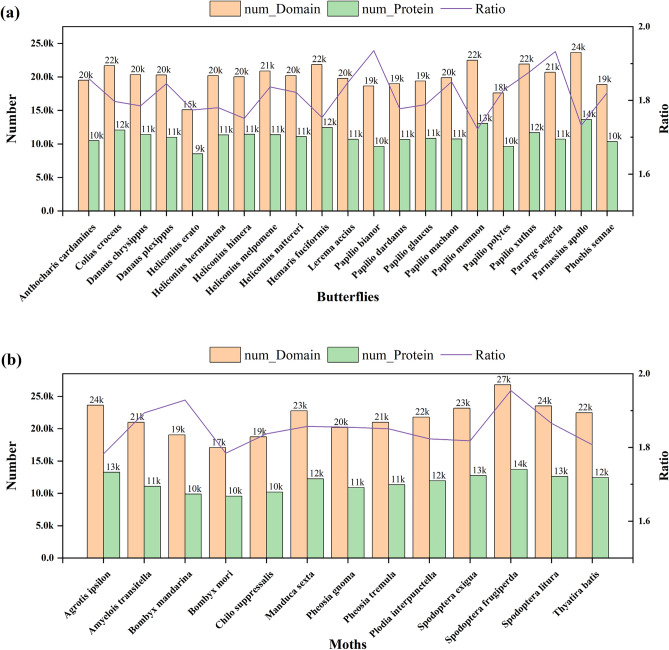
Pfam annotation results for protein sequences in 34 Lepidoptera species. (**a**) represent the Pfam annotation results for diurnal butterflies. (**b**) represent the Pfam annotation results for nocturnal moths. *num*_*Protein* and *num*_*Domain* are the number of coding genes and the number of functional domains retained in the Lepidopteran genome after Pfam annotation, respectively. *Ratio* = *num*_*Protein/num*_*Domain*, which is the (average) number of functional domains contained in a single coding gene per species.

After Pfam annotation of all protein sequences of the original 34 Lepidopteran insect species, only proteins with functional domains were retained as shown in Fig. 4.

**Figure 5:**
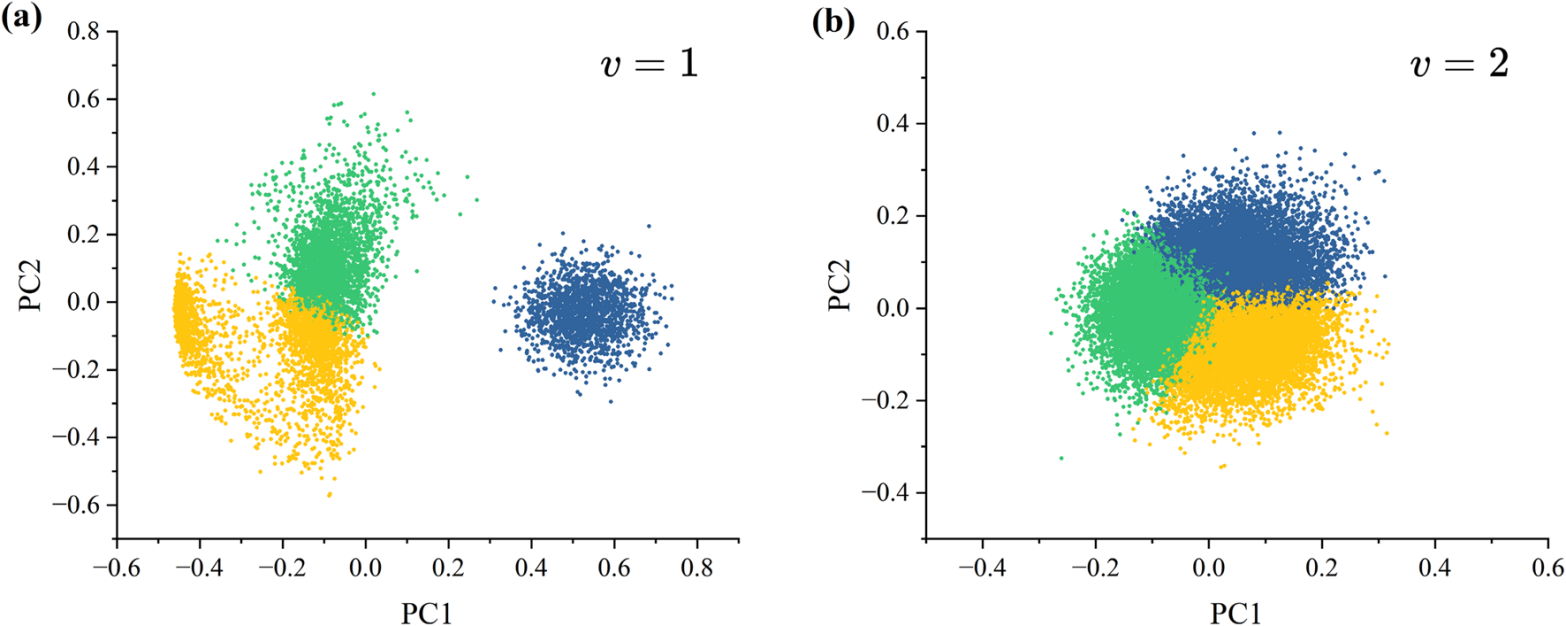
Word embedding results graph. (**a**) represents the result of word embedding when using v = 1, i.e., using the functional domain name as the word hierarchy. (**b**) represent the word embedding results of short sequences obtained as word hierarchies (v = 2) using k(k = 3) as the sliding window length.

The results of word hierarchy construction are as follows (*v* = *1*): After counting the results of Pfam annotation, there were 6,448 functional domains in all Lepidoptera proteins, and all the functional domain names were recorded as a word list. The differences in the number of kmer at different k values can lead to differences in the number of split tokens, and thus leads to differences in segmentation results. In order to reduce the occurrence of very few words to preserve the integrity of the corpus as much as possible, this paper investigates the retention of the corpus under various k-values. as shown in Supplementary-file2: Table S3. In the case of *k* = *3*, the corpus is retained to the highest degree, which is about 95% of the original corpus, so the new corpus obtained in the case of *k* = *3* is chosen for word embedding in this paper. Then, Skip-gram algorithm was used to pretrain the word embedding of the augmented corpus with an embedding dimension of 200. Then, K-means clustering was adopted to the embedded word vector (*K* = *3*, representing tripartite clusters, namely two specific taxa words, and their intersection words), and used the PCA algorithm to downscale the word vector to two dimensions as shown in Fig. 5. It is obvious from Fig. 5 that the word vectors have a clear separation trend after clustering at *v* = *1*, while they do not show a similar separation trend at *v* = *2*. This is caused by the larger scale and more obvious features of the functional domain.

### Acquisition and analysis of weights (*PAS*_*v,s*_, *GAS*_*v,T*_ and *DAS*_*v*_)

**Figure 6:**
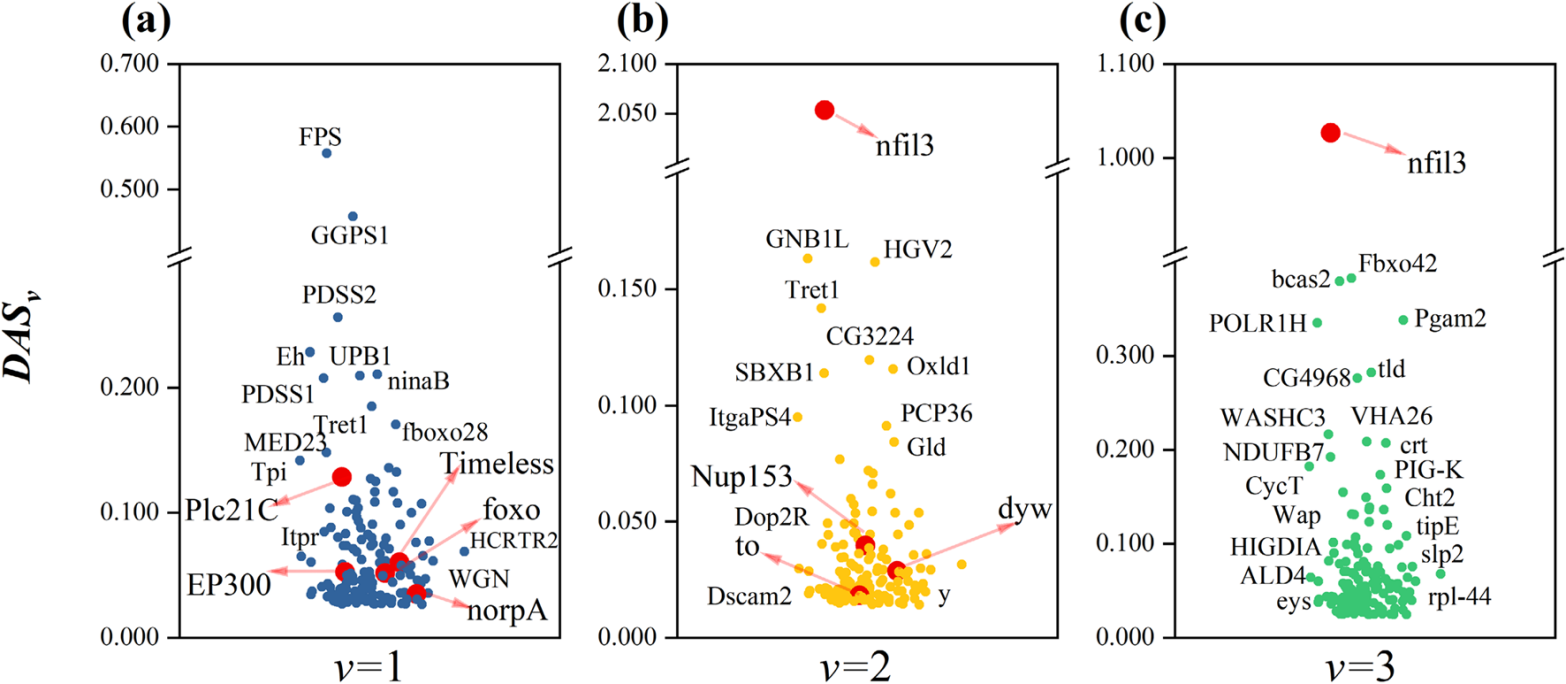
The distribution of Top 1% ${DAS}_{v}$ values based on different word hierarchies (*v*). (**a**) represent the distributions of Top 1% *DAS*_*1*_ by domain attention (v = 1); (**b**) represent the distributions of Top 1% *DAS*_*2*_ by kmer attention (v = 2); (**c**) represent the distributions of Top 1% *DAS*_*3*_ by fused attention (v = 3). The genes with red color in this figure have been reported to be associated with circadian rhythms in previous studies.

The two sets of *PAS*_*v,s*_ (*PAS*_*1,s*_ and *PAS*_*2,s*_)were obtained by inputting all protein sequences of each real species based on two word hierarchical representations corresponding to two classification models(the training process of the two models is shown in Supplementary-file1: Fig. S3) respectively. Then *PAS*_*3,s*_ was obtained by fusing *PAS*_*1,s*_ and *PAS*_*2,s*_ through the self-attention mechanism. The *GAS*_*v,T*_ obtained after clustering based on protein information annotation are shown in Supplementary-file1: Fig. S4. The distribution of top 1% *DAS*_*v*_ is shown in Fig. 6 and detail genetic information and weights at different v are available in Supplementary file 2: Tables S4–S6. Several genes with the highest *DAS*_*1*_ values were FPS, GGPS1 and ninaB, corresponding to *DAS*_*1*_ of 0.58, 0.48, 0.21, respectively. Several genes with the highest *DAS*_*2*_ values were nfil3 and GNBIL, corresponding to ${DAS}_{2}$ of 2.05 and 0.16 respectively. Several genes with the highest *DAS*_*3*_ values were nfil3 and Fbxo42, corresponding to *DAS*_*3*_ of 1.03 and 0.38 respectively. In particular, nine genes (Plc21C^39,40^ EP300^41^, Timeless^42^, foxo^43^, norpA^44^, nfil3^45^, to^46^, dyw^47^ and Nup153^48^) were found to relate to circadian rhythms in previous studies (Fig. 6a–c).

**Figure 7:**
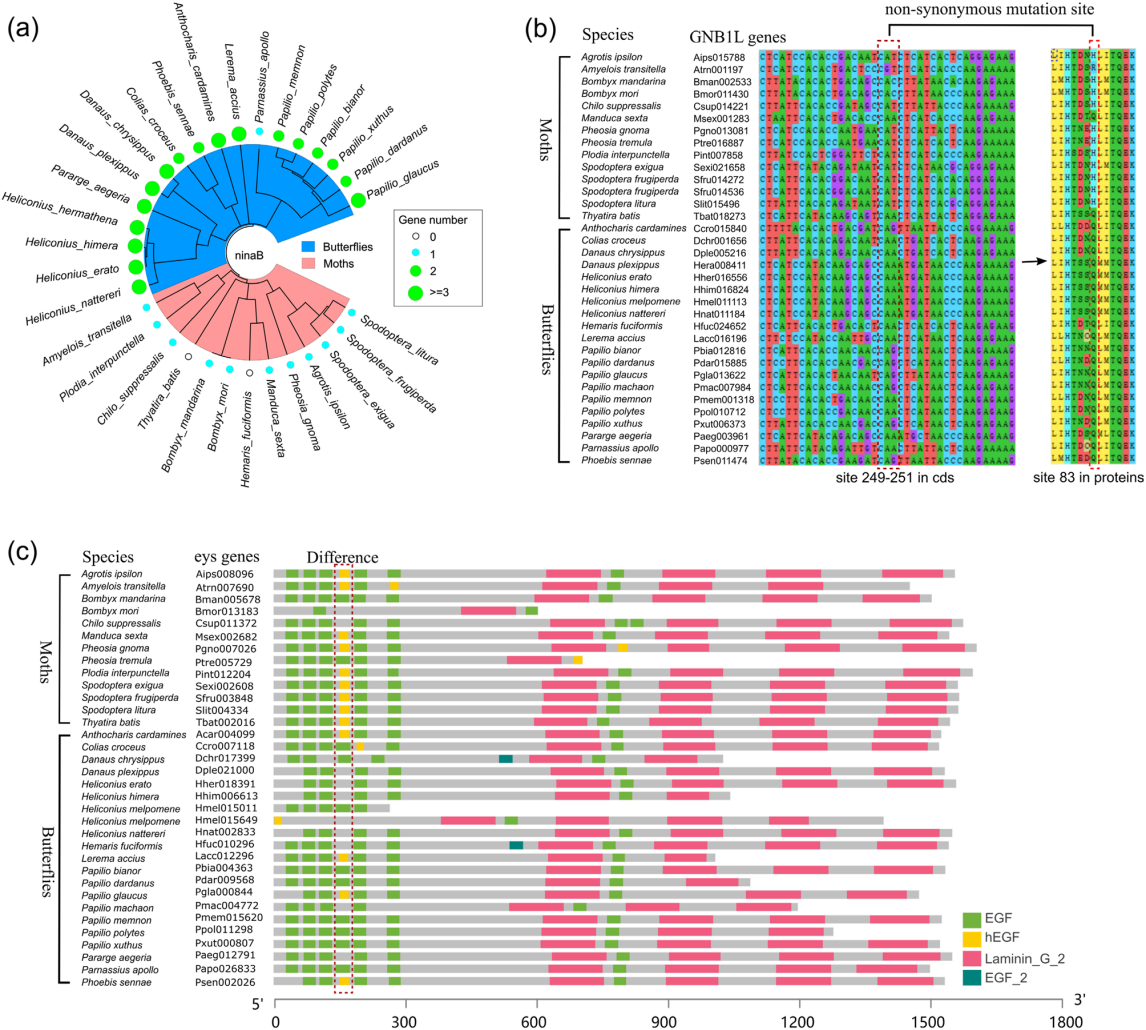
Examples of high-weight genes identified by three types of attention mechanisms. (**a**) Gene number of ninaB in butterflies and moths (identified by domain attention). (**b**) Non-synonymous mutation site of GNB1L genes in butterflies and moths (identified by kmer attention). (**c**) Domain difference of eys genes in butterflies and moths (identified by fused attention).

Furthermore, we compared the gene number of ninaB with the high *DAS*_*1*_ values (*v* = *1*) between butterflies and moths. There are more than two ninaB genes in most species of butterflies, while only one was found in each moth species (Fig. 7a). The results suggest that the method of word hierarchy (*v* = *1*) could reflect the quantity or position changes of domains, including the contraction and expansion of gene families. Meanwhile, we compared the sequence variation of GNB1l gene with the high *DAS*_*2*_ values (*v* = *2*) between butterflies and moths (Supplementary-file3). A few of specific variable sites causing the kmer weight changes were found in GNB1l gene between butterflies and moths, which contains several non-synonymous mutations (Fig. 7b). The variant kmer method of word hierarchy (*v* = *2*) could reflect the sequence variation, or small InDel in the macroevolution process of Lepidoptera. For the eys gene with the high *DAS*_*3*_ values (*v* = *3*), hEGF domain showed different insertion or deletion between butterflies and moths (Fig. 7c). These diverse domains not only affect the weight of the domain (*v* = *1*) but also the weight of the kmer (*v* = *2*), so this fusion method (*v* = *3*) may reflect the synergistic effects of the above two (v = 1 and *v* = *2*).

### KEGG enrichment of key genes with high weights

**Figure 8:**
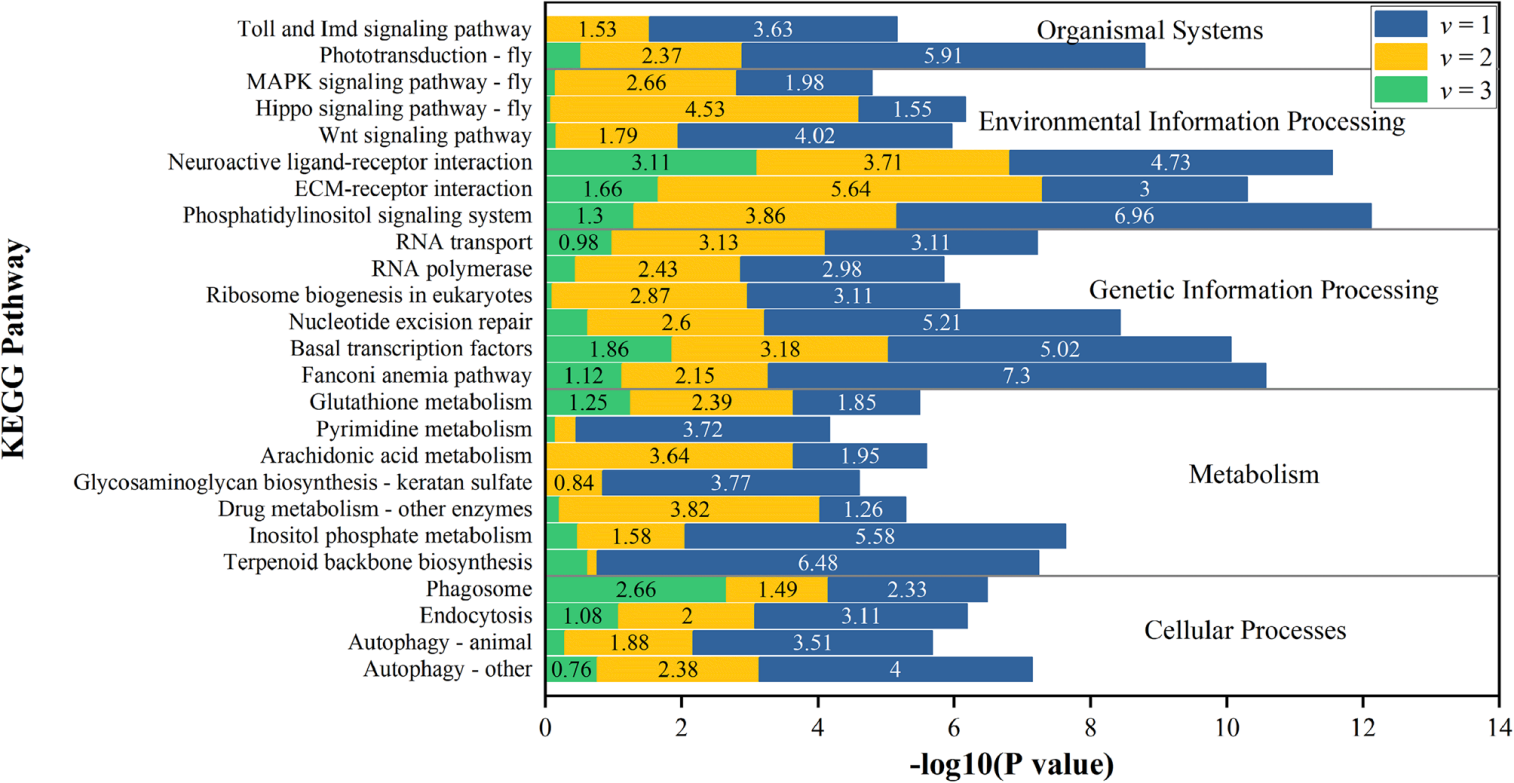
KEGG enrichment analysis results of high-weight genes (top 1%). The reference species is silkworm. Blue color stands for the domain attention; Yellow color stands for the kmer attention; Green color stands for the fused attention.

The above genes (Top 1%) obtained by three types of attention mechanisms (domain attention, kmer attention and fused attention) were regarded as the key genes in macroevolution of Lepidoptera. These genes were taken for the KEGG enrichment (as shown in Fig. 8). It can be seen that the three groups of genes have some commonality and are all enriched in some specific pathways, such as *Phototransduction—fly* (where Negative logarithmic P-value is 5.91, 2.37, 0.52, respectively, for a total of 8.80), *Phosphatidylinositol signaling system* (where Negative logarithmic P-value is 6.96, 3.86, 1.30, respectively, for a total of 12.12), *Drug metabolism—other enzymes* (where Negative logarithmic P-value is 10.695, 4.588, 0.21, respectively, for a total of 5.29), *Fanconi anemia pathway* (where Negative logarithmic P-value is 7.3, 2.15, 1.12, respectively, for a total of 10.57), *Terpenoid backbone biosynthesis* (where Negative logarithmic P-value is 6.48, 0.14, 0.62, respectively, for a total of 7.24), etc.

## Discussion

Phenotypic differences of macroevolution usually represent the synergistic action of multiple key genes in evolutionary biology^49–51^. However, there are still some challenges to establish a universal method or model for exploring these key genes of macroevolution^49,52^. The first challenge comes from the diversity of biological sequences (DNA and proteins). A central issue for machine learning methods is how to design a good representation for the biological sequences^53,54^. The word embeddings can capture the semantic correlation between words and reflect the contextual relationship of the original sequence. We used two characteristic word-level construction methods including functional domain (*v* = *1*) and variable length kmer (*v* = *2*). The functional domain embedding can well reflect the domain variations at a large scale, such as gene duplication^15,16^, structure variation^19^, etc. The kmer embedding can well reflect the sequence variation, such as selective evolution^17,18^, small InDel, etc. Instead of the common kmer method^55–58^, the variable length kmer word embedding comprehensively consider the kmer with different lengths based on probability. The embedding and partitioning method can better reserve the diversity of gene sequences. Therefore, these two methods of word embedding take into account various scales of gene variation as well as various relationships with biological sequence location and context. In addition to the word hierarchies at both scales (*v* = *1* and *v* = *2*), a fusion method (*v* = *3*) is proposed to capture the combined molecular mechanisms influenced by domain and variant kmer. The second challenge is to clarify the inference process of DL algorithms^59^. This study focuses on the molecular mechanisms behind macroevolution and models it into a computer classification problem using the genomes of taxa that have undergone macroevolution. The aim is not just to create a classification model with high accuracy, but to understand the inference process itself, specifically which genes are important for classification. To achieve this, the study proposes the inclusion of AM as a feasible strategy^60^. Combining with the hierarchical structural properties of biological sequences, this study incorporates a hierarchical AM into the deep classification model, so that the model can focus not only on important “words” (Domain/short sequences) but also on important “sentences” (proteins). Interestingly, three types of attention mechanisms (domain attention, kmer attention and fused attention) maybe stand for different molecular mechanisms of macroevolution in evolutionary biology (Fig. 7).

Previous studies indicated that the two major taxa of butterflies and moths showed significant differences in circadian rhythm^61^. Our results identified a number of genes with high weights, which were mainly enriched in Phototransduction—fly, Phosphatidylinositol signaling system, Inositol phosphate metabolism, Wnt signaling pathway, MAPK signaling pathway—fly, Notch signaling pathway as well as FoxO signaling pathway etc. Most of these genes have been reported to be associated with the control of circadian rhythms in insects, such as dyw (daywake)^47^, to (takeout)^46^, EP300 (E1A binding protein p300)^41^, Plc21C (Phospholipase C at 21C)^39,40^, norpA (no receptor potential A)^44^, Nup153 (Nucleoporin 153kD)^48^, Nfil3 (nuclear factor, interleukin 3, regulated)^45^, foxo (forkhead box, sub-group O)^43^ and Timeless (timeless circadian regulator)^42^ (Figs. 6, 7). These reported circadian rhythm-related genes with high weights proved the validity of our method. Moreover, it is suggested that some of the other high-weight genes identified in this paper may also play important roles in the macroevolution of Lepidopterans. We found some high weights genes were reported to be associated with senses in Lepidopteran insects^18,62^, such as ninaB (neither inactivation nor afterpotential B)^63^, eys(eyes shut)^64^, and Dscam2 (Down syndrome cell adhesion molecule 2)^65^ related to axonal tiling of the insect visual system, the aop (anterior open) gene related to the photoreceptor rhabdomere^66^, Itpr (Inositol 1,4,5,-trisphosphate receptor) related to visual and olfactory transduction^67^, as well as WGN (Wengen) related to photoreceptor cell axon guidance^68^. Additionally, we also identified many genes that may be involved in these behavioral differences in butterflies and moths, for example, y (yellow) and Dop2R (Dopamine 2-like receptor) are involved in male courtship behavior of insects^69,70^, HCRTR2(hypocretin receptor 2) may be involved in regulating feeding and sleep behavior^71,72^. The above genes related to circadian rhythms, sensory organs, and behavioral habits should help us to explain the macroscopic differences of diurnal butterflies and nocturnal moths in Lepidoptera.

## Conclusion

This paper proposes a new method for identifying the important genes of macroevolution using deep learning and attention mechanism. Based on this new method, we mined a few of key genes related to the phenotypic differences (circadian rhythms, sensory organs, as well as behavioral habits etc) of diurnal butterflies and nocturnal moths in Lepidoptera. It not only provides a novel method to identified the key genes of macroevolution at the genomic level, but also helps us to understand the microevolution mechanisms of diurnal butterflies and nocturnal moths in Lepidoptera.

### Supplementary Information

Supplementary Figures.

Supplementary Tables.

Supplementary Information.

## Data Availability

The source data and experiment code for our implementation are available for public access and can be found in GitHub (https://github.com/JiaweiMao12135/IKGM). The code is written in Python and serves as a reference for the experiments conducted in this paper. We encourage collaboration and feedback from the community to improve the code and foster future advancements. Feel free to report any issues or suggest improvements by creating an issue in the GitHub repository's issue tracker. Please note that while we have taken measures to thoroughly test the code, unforeseen issues or limitations may still exist. We appreciate your understanding and assistance in refining the codebase. By sharing our code, we aim to contribute to the open research community and promote reproducibility, allowing others to validate our results and build upon our work.
