# Supplementary material for: A novel method for identifying key genes in macroevolution based on deep learning with attention mechanism
Source: Sci Rep. 2023 Nov 13;13:19727. doi: 10.1038/s41598-023-47113-9 (PMC10643560; doi:10.1038/s41598-023-47113-9)
Supplement: Supplementary file 1 — Supplementary Figures. [file 41598_2023_47113_MOESM1_ESM.docx]

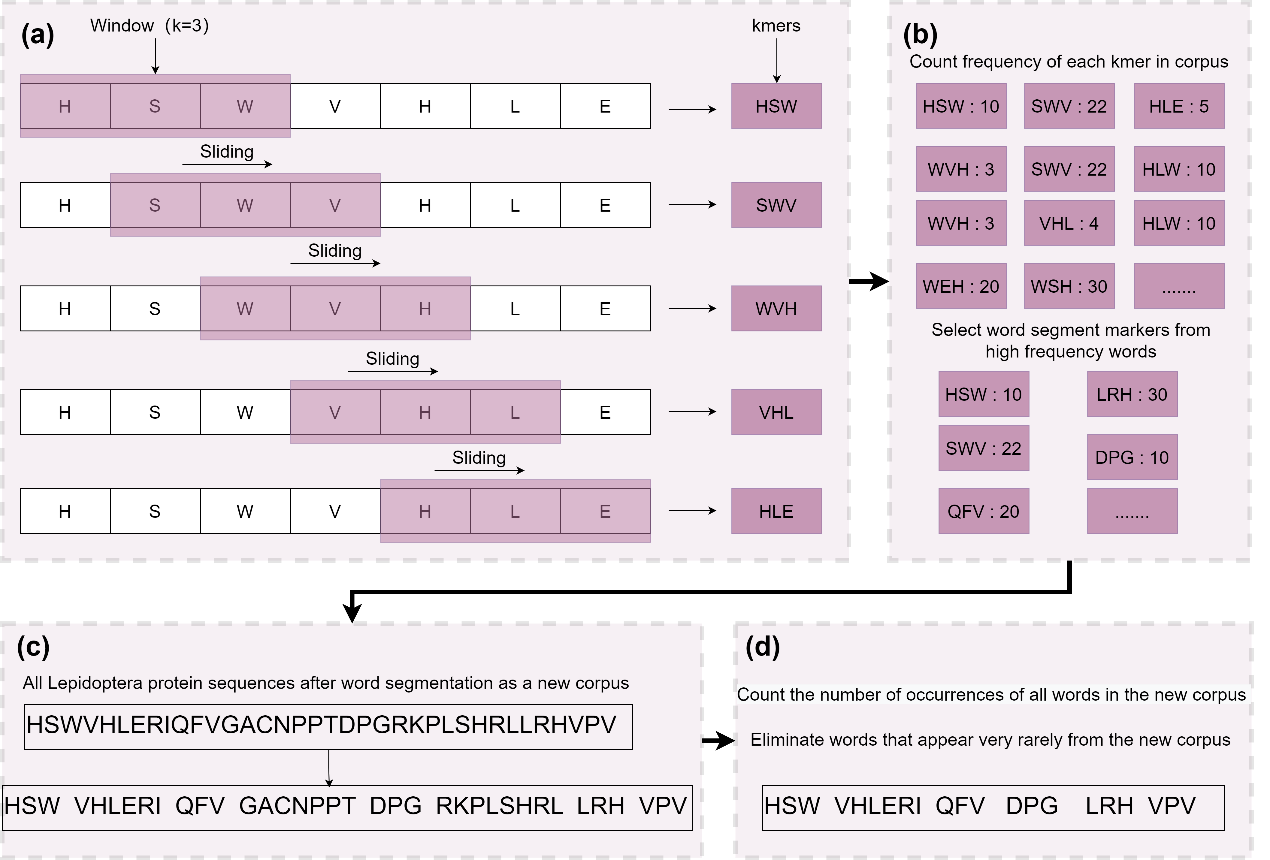


**Figure S1. The generation process of variant kmer.** (a) A sliding window of window size k (k=3) was used for all protein sequences to obtain multiple kmer. (b) The frequencies of all kmer occurrences in protein sequences(corpus) were counted, and the kmer with frequencies greater than the upper quartile were selected as the segment markers. (c) The corpus is segmented using these segmentation markers and all the short sequences of unequal length obtained after segmentation are used as words. (d) All words were counted for word frequency, and the very low frequency words were eliminated, and the retained words were used to characterize the corpus.

**Figure S2. Schematic representation of importance (weights) in the classification process.** (a) The words in red in the example protein sequences in the figure are clearly different in the two taxa, then they should be more important for the classification of the taxa. (b) The protein (sentence) in red in the example Genome in the figure are significantly different in the two taxa, then they should be more important for the classification of the taxa.


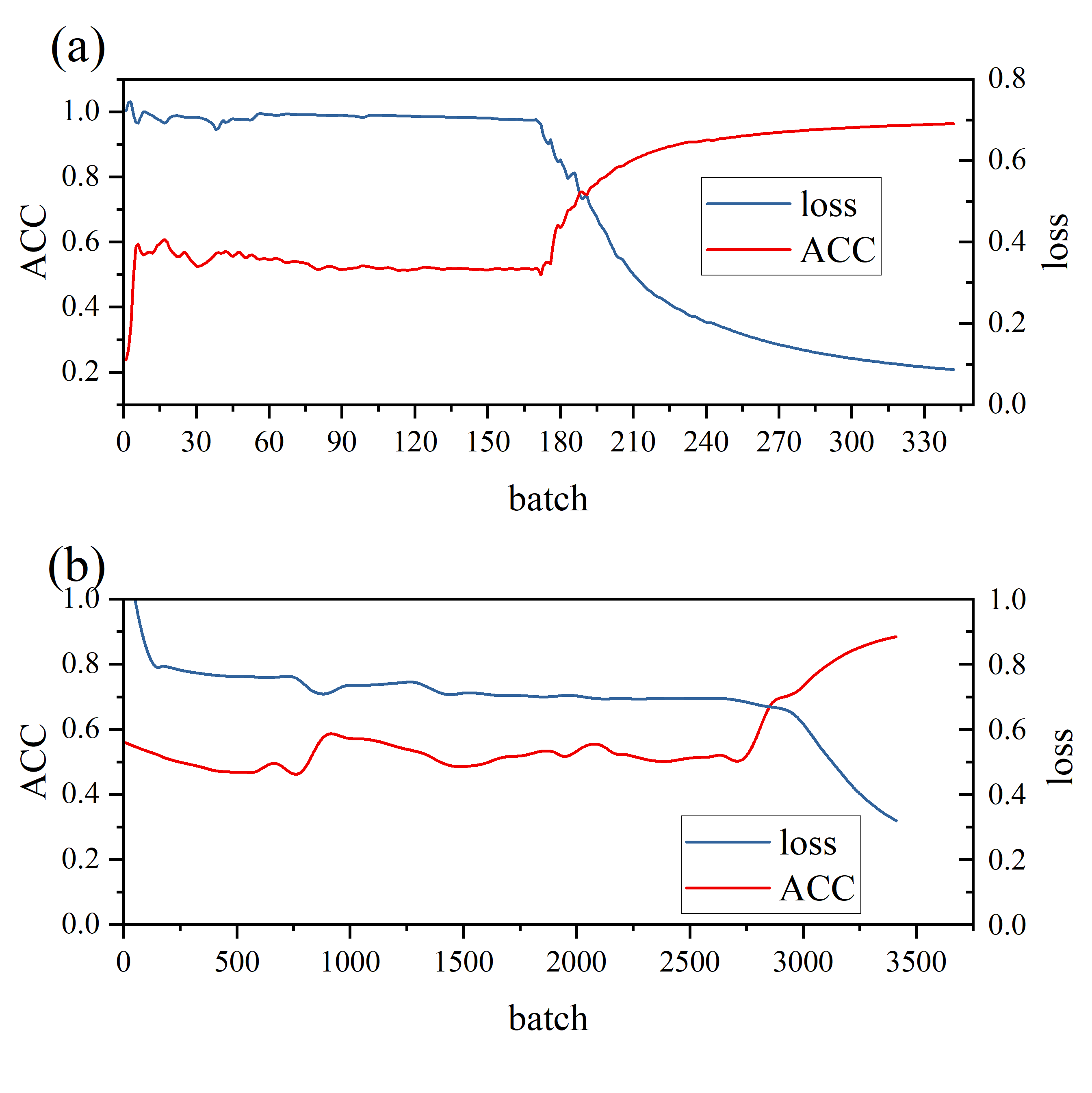


**Figure S3. Variation in accuracy and loss during training using hierarchical attention classification networks.** (a) represents the training process of constructing a dataset of word hierarchies using functional domain names(v=1). (b) represents the training process of constructing a dataset of word hierarchies using functional variant kmer(v=2).


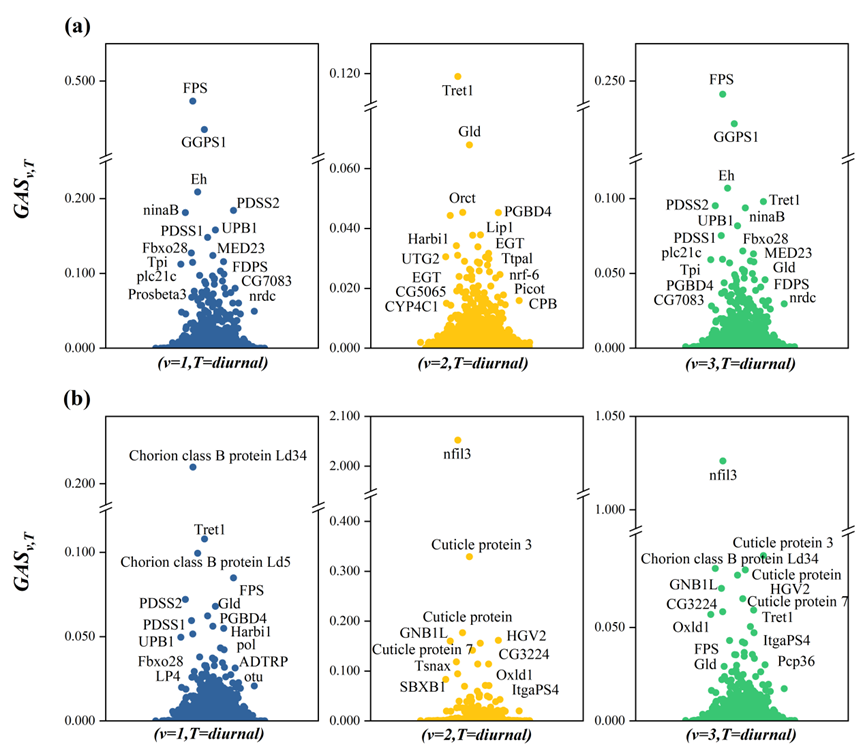


**Figure S4. The distribution of** $\boldsymbol{GAS}_{\boldsymbol{v}\boldsymbol{,}\boldsymbol{T}}$ **values under different** $\boldsymbol{v}$**.** (**a**) Distribution of ${GAS}_{v,T}$ of diurnal lepidopteran taxa *(*$T=diurnal$*)*; (**b**) Distribution of ${GAS}_{v,T}$ of diurnal lepidopteran taxa *(*$T=diurnal$; In addition, the ${GAS}_{v,T}$ are normalized results.
