# Supplementary material for: A novel method for identifying key genes in macroevolution based on deep learning with attention mechanism
Source: Sci Rep. 2023 Nov 13;13:19727. doi: 10.1038/s41598-023-47113-9 (PMC10643560; doi:10.1038/s41598-023-47113-9)
Supplement: Supplementary file 3 — Supplementary Information. [file 41598_2023_47113_MOESM3_ESM.pdf]

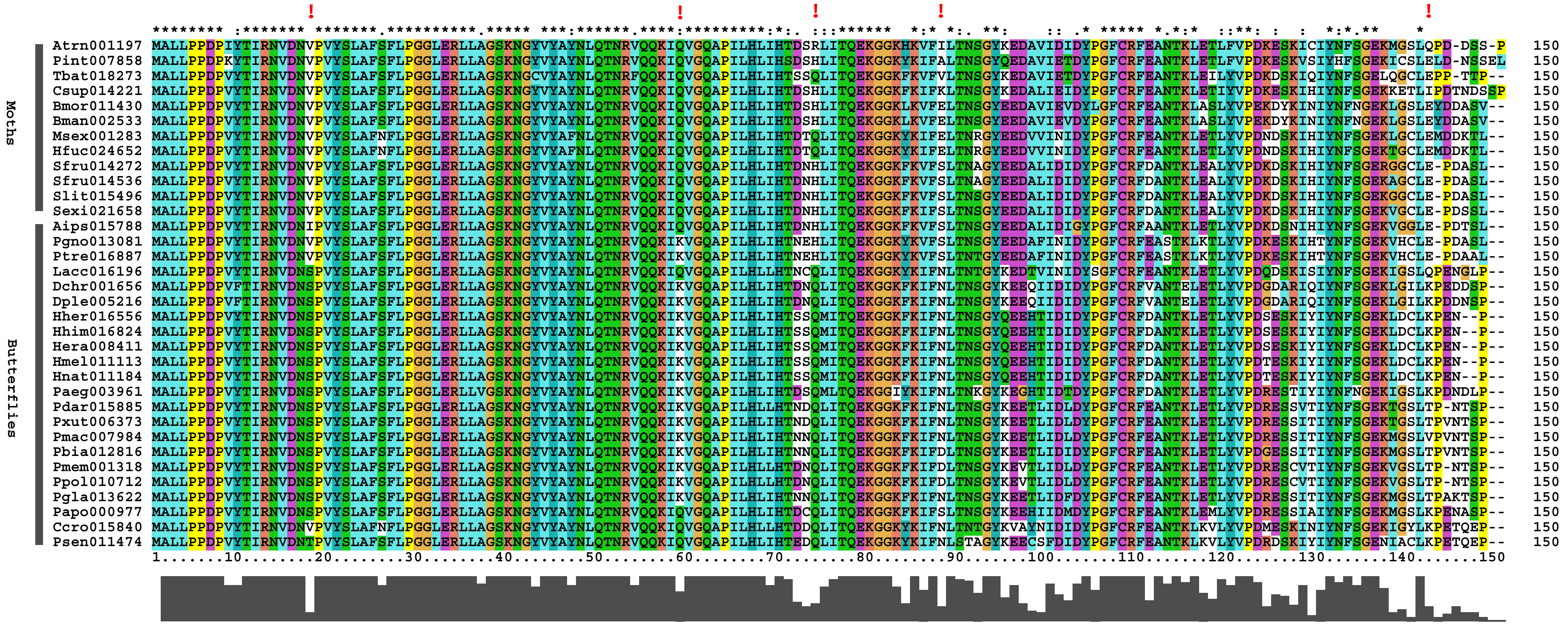

Symbol(!) stands for the variable site of GNB1l gene between butterflies and moths.

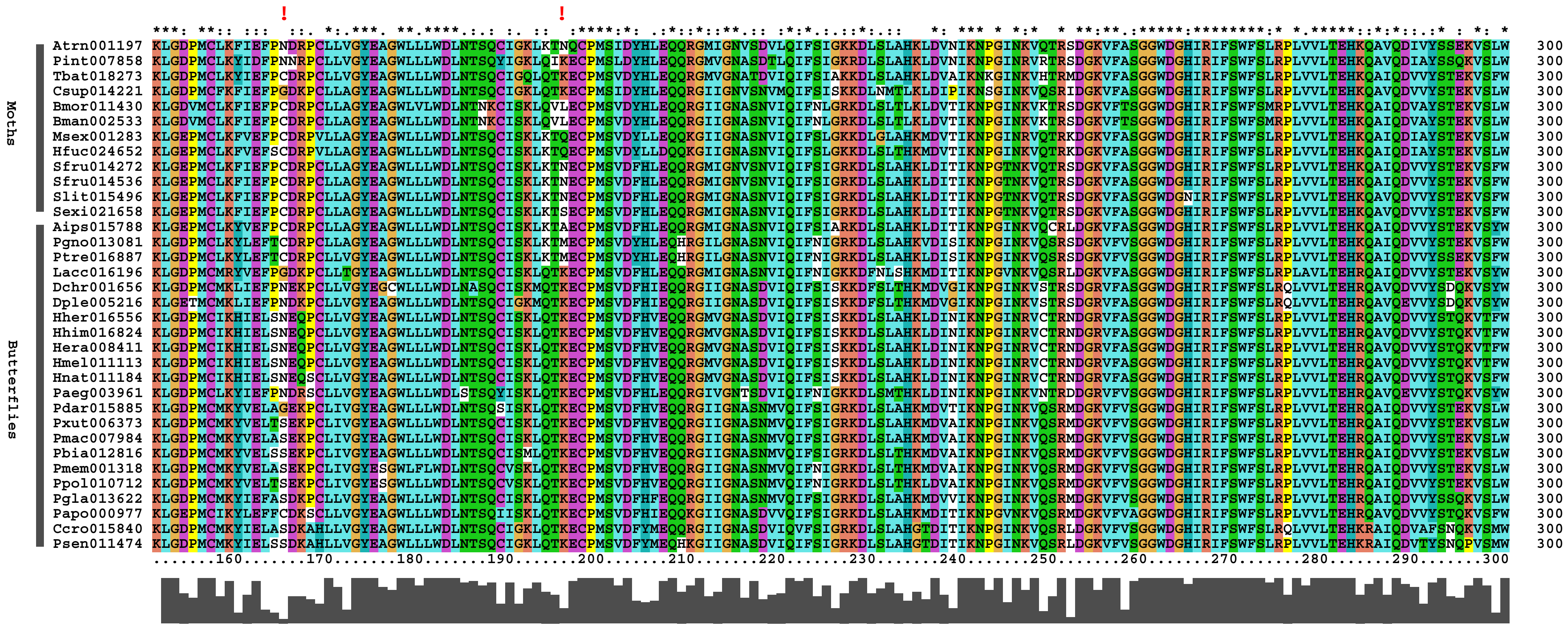

Symbol(!) stands for the variable site of GNB1l gene between butterflies and moths.

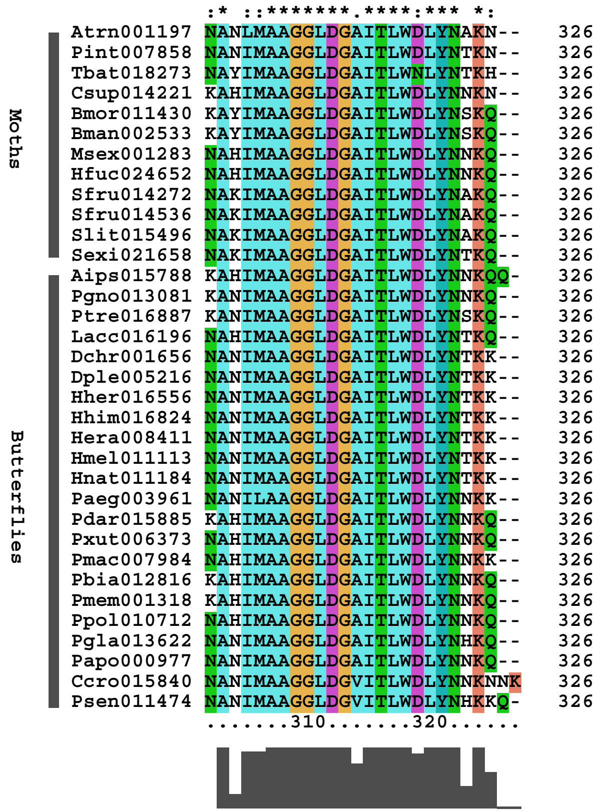
:* ::********.****:*** *:
Moths
Butterflies
Atrn001197 NANLMAAGGLDGAITLWDLYNAKN-- 326
Pint007858 NANIMAAGGLDGAITLWDLYNTKN-- 326
Tbat018273 NAYIMAAGGLDGAITLWNLYNTKH-- 326
Csup014221 KAHIMAAGGLDGAITLWDLYNNKN-- 326
Bmor011430 KAYIMAAGGLDGAITLWDLYNSKQ-- 326
Bman002533 KAYIMAAGGLDGAITLWDLYNSKQ-- 326
Msex001283 NAHIMAAGGLDGAITLWDLYNNKQ-- 326
Hfuc024652 NAHIMAAGGLDGAITLWDLYNNKQ-- 326
Sfru014272 NAKIMAAGGLDGAITLWDLYNAKQ-- 326
Sfru014536 NAKIMAAGGLDGAITLWDLYNAKQ-- 326
Slit015496 NAKIMAAGGLDGAITLWDLYNAKQ-- 326
Sexi021658 NAKIMAAGGLDGAITLWDLYNTKQ-- 326
Aips015788 KAHIMAAGGLDGAITLWDLYNNKQQ- 326
Pgno013081 KANIMAAGGLDGAITLWDLYNNKQ-- 326
Ptre016887 KANIMAAGGLDGAITLWDLYNSKQ-- 326
Lacc016196 NAHIMAAGGLDGAITLWDLYNTKQ-- 326
Dchr001656 NANIMAAGGLDGAITLWDLYNTKK-- 326
Dple005216 NANIMAAGGLDGAITLWDLYNTKK-- 326
Hher016556 NANIMAAGGLDGAITLWDLYNTKK-- 326
Hhim016824 NANIMAAGGLDGAITLWDLYNTKK-- 326
Hera008411 NANIMAAGGLDGAITLWDLYNTKK-- 326
Hmel011113 NANIMAAGGLDGAITLWDLYNTKK-- 326
Hnat011184 NANIMAAGGLDGAITLWDLYNTKK-- 326
Paeg003961 NANILAAGGLDGAITLWDLYNNKK-- 326
Pdar015885 KAHIMAAGGLDGAITLWDLYNNKQ-- 326
Pxut006373 NAHIMAAGGLDGAITLWDLYNNKQ-- 326
Pmac007984 NAHIMAAGGLDGAITLWDLYNNKK-- 326
Pbia012816 KAHIMAAGGLDGAITLWDLYNNKQ-- 326
Pmem001318 KAHIMAAGGLDGAITLWDLYNNKQ-- 326
Ppol010712 NAHIMAAGGLDGAITLWDLYNNKQ-- 326
Pgla013622 NANIMAAGGLDGAITLWDLYNHKQ-- 326
Papo000977 NANIMAAGGLDGAITLWDLYNNKQ-- 326
Ccro015840 NANIMAAGGLDGVITLWDLYNNKNNK 326
Psen011474 NANIMAAGGLDGVITLWDLYNHKKQ- 326
.......310.......320......
